# RIP140-Mediated NF-κB Inflammatory Pathway Promotes Metabolic Dysregulation in Retinal Pigment Epithelium Cells

**DOI:** 10.3390/cimb44110393

**Published:** 2022-11-21

**Authors:** Zeli Guo, Yuli Shen, Jianwen Zhong, Zhuoyun Li, Qi Guo, Xiangchao Yao, Yandong Wang, Wenyu Wu

**Affiliations:** State Key Laboratory of Ophthalmology, Zhongshan Ophthalmic Center, Sun Yat-sen University, Guangdong Provincial Key Laboratory of Ophthalmology and Visual Science, Guangzhou 510060, China

**Keywords:** RIP140, NF-κB, metabolism, inflammation, age-related macular degeneration

## Abstract

Metabolic dysregulation of the retinal pigment epithelium (RPE) has been implicated in age-related macular degeneration (AMD). However, the molecular regulation of RPE metabolism remains unclear. RIP140 is known to affect oxidative metabolism and mitochondrial biogenesis by negatively controlling mitochondrial pathways regulated by PPAR-γ co-activator-1 α(PGC-1α). This study aims to disclose the effect of RIP140 on the RPE metabolic program in vitro and in vivo. RIP140 protein levels were assayed by Western blotting. Gene expression was tested using quantitative real-time PCR (qRT-PCR), ATP production, and glycogen concentration assays, and the release of inflammatory factors was analyzed by commercial kits. Mice photoreceptor function was measured by electroretinography (ERG). In ARPE-19 cells, RIP140 overexpression changed the expression of the key metabolic genes and lipid processing genes, inhibited mitochondrial ATP production, and enhanced glycogenesis. Moreover, RIP140 overexpression promoted the translocation of NF-κB and increased the expression and production of IL-1β, IL-6, and TNF-α in ARPE-19 cells. Importantly, we also observed the overexpression of RIP140 through adenovirus delivery in rat retinal cells, which significantly decreased the amplitude of the a-wave and b-wave measured by ERG assay. Therapeutic strategies that modulate the activity of RIP140 could have clinical utility for the treatment of AMD in terms of preventing RPE degeneration.

## 1. Introduction

Age-related macular degeneration (AMD) is the most common form of retinal degeneration and is a multifactorial disease that affects the macular region of the retina. Due to population aging, the number of AMD patients is expected to rise to 196 million by 2020 and to 288 million by 2040 [1]. As AMD disease progresses, there are two results, one is the dry form (geographic atrophy), and the other is the wet form (neovascular AMD). In both subtypes of AMD, the retinal pigment epithelium plays a crucial role in the pathogenesis of AMD [2]. RPE cells are monolayer cells, which mainly play a photosensitive role and form a blood–eye barrier. RPE cells have high metabolic demands because they need to maintain photoreceptor outer-segment synthesis and phagocytosis, retinoid recycling, and maintenance of membrane potential and ion flux through Na^+^ K^+^-ATPases [3]. It is becoming more evident that the metabolic state of the retina profoundly influences the occurrence and development of AMD. The main energy source of retinal photoreceptor cells is glucose, which is provided by the choriocapillaris through Bruch’s membrane (BrM) and RPE cells. The retina and RPE have different metabolic functions, which mainly maintain their respective ecosystems both in vivo and in vitro [4]. It is reported that RPE also acts as a blood–outer retinal barrier transporting nutrients, including glucose, to photoreceptors, and inhibition of RPE glucose transport to photoreceptors causes photoreceptor starvation [5]. Studies indicate several mechanisms involved in AMD, such as elevated oxidative stress, altered mitochondrial bioenergetics, dysregulated RPE metabolism, and increased inflammation [6,7,8]. Therefore, the main hypothesis is that the downregulation of the metabolic level is the main cause of retinal diseases. Therefore, approaches to enhance energy metabolism will have a therapeutic benefit on retinal diseases such as AMD.

A well-studied transcription coactivator, PPARγ coactivator-1a (PGC-1a), regulates the expression of metabolic genes associated with mitochondrial and metabolic adaptations. It has been indicated that PGC-1α plays mutable roles in inducing oxidative phosphorylation (OXPHOS), fatty-acid gene and protein expression, regulating oxidative metabolism, and fatty-acid beta-oxidation in the RPE [9]. PGC-1α also plays an important role in maintaining the homeostatic state of RPE, such as glucose metabolism, autophagy, and epithelial integrity [10]. It is worth noting that PGC-1α was decreased in AMD-derived RPE cells [11]. A corepressor for nuclear receptors and transcription factors, named receptor-interacting protein 140 (RIP140), downregulates many catabolic pathways, such as oxidative phosphorylation, tricarboxylic acid cycle, glycolysis, and fatty acid oxidation [12]. Many genes are regulated both by RIP140 and PGC-1a, but RIP140 works as a corepressor and inhibits energy utilization in cardiomyocytes [13]. However, the precise function of RIP140 in RPE cells is incompletely understood. In addition, RIP140 was found to function in macrophages as a coactivator for NF-κB to modulate the production of proinflammatory cytokines such as TNF, IL-1β, and IL-6 [14]. However, the effects of RIP140 in RPE cells remain to be elucidated. Owing to the importance of the metabolism and inflammation status of RPE cells in the development of AMD, we aim to study the effects of RIP140 on the energy state and the crosstalk between metabolic and inflammation pathways in RPE cells in this study.

## 2. Materials and Methods

### 2.1. Cell Culture

ARPE-19, a human RPE cell line, was obtained from American Type Culture Collection. The results of short tandem repeat (STR) showed that the ARPE-19 sample was a 100% match to the ATCC ARPE-19 cell line. Cells were in a 1:1 mixture of Dulbecco’s Modified Eagle’s and Ham’s F12 media, supplemented with 10% FBS and maintained in a 95% air and 5% CO_2_ humidified atmosphere at 37 °C.

### 2.2. Recombinant Adenovirus Preparation

Adenovirus expressing RIP140 (Ad-RIP140) or Ad-GFP were gifts from Dr. Yanfang Chen (Department of Pharmacy, The Second Affiliated Hospital of Guangzhou Medical University, Guangzhou, China). Adenovirus was amplified and purified using OBiO Technology (Shanghai, China) and then titrated and diluted to a suitable titer before subretinal injection.

### 2.3. Adenovirus Infection and siRNA Interference

Cells were exposed to adenovirus expressing RIP140-specific or nonspecific control infected for 48 h before RNA or protein extracts. siRNA against human-specific p65-NF-κB (#6261) was purchased from CST. Cells were incubated for 48 h in transfection reagents containing 100 nmol siRNA duplexes and other additions as indicated in the figures according to the manufacturer’s instructions.

### 2.4. Mitochondrial Activity Assay

ATP levels were used to assay the mitochondrial activities. Samples were incubated for 2 h with 10 μM of bromopyruvate analog (3-BrPA) (Millipore, Darmstadt, Germany, cat# 376817), an inhibitor of glycolytic hexokinase II enzyme. Measurements were performed according to the manufacturer’s instructions (Promega, cat# G8000, Fitchburg, WI, USA).

### 2.5. Western Blotting Assay

After various treatments, ARPE-19 cells were washed twice with PBS and lysed in RIPA buffer containing protease and phosphatase inhibitor cocktails (MERCK, Rahway, NJ, USA). Whole-cell lysates of cultured cells were prepared by scraping cells into RIPA buffer. The resulting cell lysates were clarified by centrifugation at 12,000× *g* for 15 min at 4 °C. Equal amounts of protein from each sample were separated by SDS–PAGE and transferred to PVDF membrane (Amersham Pl. Little Chalfont, Buckinghamshire, UK). After incubation with corresponding antibodies overnight.All the antibodies were purchased from Cell Signaling Technology Inc. (Danvers, MA, USA), the membranes were visualized using the Dura detection system (Thermo Fisher Scientific, Waltham, MA, USA).

### 2.6. Cytoplasmic Glycogen Concentration Assay

Cytoplasmic glycogen levels were assayed using the Glycogen Assay Kit (Sigma, Merck KGaA, Darmstadt, Germany, cat# MAK016) according to the manufacturer’s instructions.

### 2.7. Isolation of Cytoplasmic and Nuclear Proteins

Cytoplasmic and nuclear proteins were extracted using NEPER Nuclear and Cytoplasmic Extraction Reagents from Thermo Scientific (Waltham, MA USA), according to the manufacturer’s instructions.

### 2.8. Quantitative Real-Time PCR (qRT-PCR)

Total RNA was extracted using TRIzol (Invitrogen, Carlsbad, CA, USA) according to the manufacturer’s instructions. RNA was reverse transcribed at 37 °C for 60 min in a 10 μL reaction mixture using the Reverse Transcription System from Promega (Fitchburg, WI, USA). cDNA was synthesized and amplified using a SuperScript-III kit (Takara, Dalian, China). Primers used for qPCR are listed in Table S1 in the Supplemental Data.

Previously described primers were employed for the genes amplification list below: ACADM, ACADS, ATP5O, COX4I1, COX5B, HADHB, ERRα, NRF-1, and PPARα [9], ApoA1 [15], ApoB [16], ApoE [17], PPARγ [7], PPARβ [18], Glu1 [19], Glu4 [20], and LDLR, GAPDH [21].

### 2.9. ELISA for TNF-α and IL-1β, IL-6 Measurement

The culture medium was collected after treatment and centrifuged at 600× *g* for 5 min to pellet the cell debris. The supernatant was removed and stored at −80 °C prior to analysis. TNF-a levels in the supernatant were determined with sandwich ELISA using the dual antibody kits (R&D Systems) according to the manufacturer’s instructions and expressed as pg mL^−1^.

### 2.10. Animals

Male Sprague–Dawley rats (7–8 weeks old, 230–300 g body weight) were used in the present study. All animal procedures performed in our study complied with the ARVO Statement for the Use of Animals in Ophthalmic and Vision Research and all protocols and regulations established by the Animal Care and Use Committee of Zhongshan Ophthalmic Center, Sun Yat-Sen University. A heating pad and a heating lamp were used to maintain the rectal temperature of the rats at 37 °C during experiments.

### 2.11. Subretinal Injection

For subretinal injections, the animals were anesthetized by intraperitoneal injection of zolazepam and tiletamine (12.5 mg/kg). Adenoviral vectors of Ad-GFP (as a negative control) or Ad-RIP140 (5 × 10^9^ PFU/eye) were injected subretinally in a volume of 5 μL into the right eye of each animal as described previously [22]. The contralateral eye was not injected. After three days of adenoviral vectors injected, an inhibitor of NF-κB BAY 11-7082 (500 pmol/eye) was injected subretinally, as described previously [23].

### 2.12. Electroretinogram (ERG)

Scotopic ERG analysis was used to measure the loss of rod function. Rats were dark-adapted overnight. The following day the rats were anesthetized by intraperitoneal injection of zolazepam and tiletamine (12.5 mg/kg). The pupils were dilated with compound tropicamide eye drops (Santen Pharmaceuticals Co. Ltd., Osaka, Japan). Ophthalmic anesthesia was induced with 0.5% tetracaine hydrochloride eye drops (Zhongshan Ophthalmic Center, Sun Yat-Sen University), and hypromellose eye drops (Zhongshan Ophthalmic Center, Sun Yat-Sen University) were applied to increase the electrical conductivity of rat eyes. Gold wire electrodes were placed over the corneas of the anesthetized rat, the reference electrode was placed in the mouth, and the grounding needle electrode was placed in the tail. Roland Consult visual electric physiological system and Color Ganzfeld Q450C stimulator were used for FERG, which obtained parameters including scotopic 0.01 ERG, scotopic 3.0 ERG, scotopic 10.0 ERG, scotopic 3.0 oscillatory potential ERG, and photopic 3.0 ERG. After the test was completed, the a- and b-wave amplitudes and the a- and b-wave peak times were recorded in each group. The b-wave was measured from the absolute peak of the a-wave to the peak of the positive deflection within 2000 ms of the flash stimulus.

### 2.13. Statistical Analysis

Results are expressed as mean values ± SD unless specified. Statistical analysis was performed with one-way analysis of variance (ANOVA) or Student’s *t*-test. A *p*-value of 0.05 was considered significant.

## 3. Results

### 3.1. RIP140 Regulates the Key Metabolic Genes and Lipid Processing Genes in ARPE-19 Cells

The RIP140/PGC-1α axis modulates mitochondrial function. Crucially, RIP140 negatively regulates PGC-1α as a corepressor [24]. In the RPE cells, PGC-1a regulates oxidative metabolism and fatty-acid beta-oxidation and also induces the genes of oxidative phosphorylation (OXPHOS), fatty-acid [9], while the cellular energy state and mitochondrial biogenesis were regulated by RIP140 and PGC-1α, contrary to a previous study [25]. To study the role of RIP140 in RPE cells, we first tested the relative gene expression in RPE cells overexpressing RIP140.

**Figure 1:**
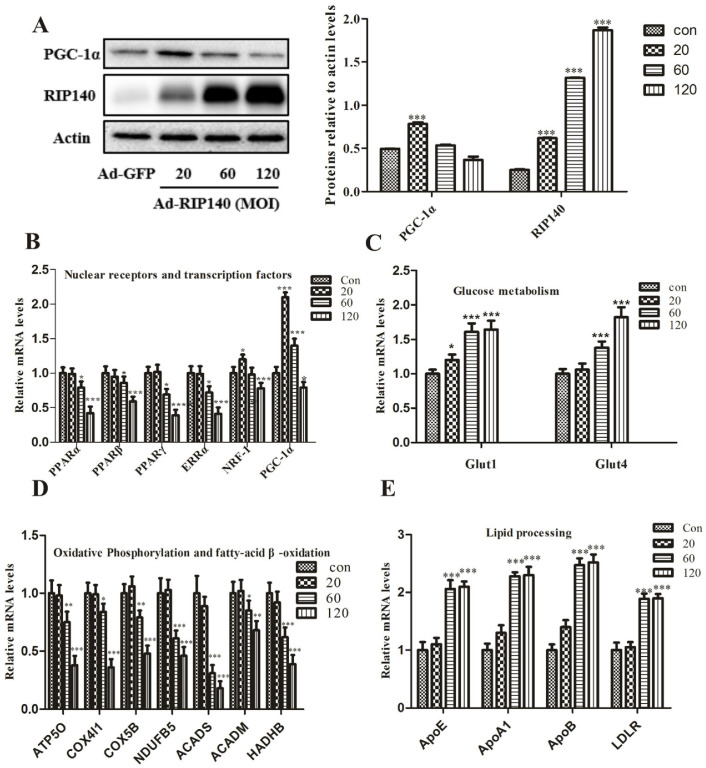
Key metabolic genes and lipid processing genes regulated by adenovirus-mediated overexpression of RIP140 in a dose-dependent manner. (**A**) RIP140 and PGC-1α expression detected by Western blot analysis after Ad-RIP140 infection. (**B**) Results of real-time RT-PCR analysis for transcriptional regulators in RPE cells infected with Ad-RIP140 (**C**,**D**) Expression of metabolic genes associated with glucose and fatty acid metabolism at mRNA levels in the presence of different expression of RIP140. (**E**) Lipid processing genes altered in RIP140 groups. * *p* < 0.05, ** *p* < 0.01, *** *p* < 0.001 vs. Ad-GFP group.

RIP140 overexpression markedly upregulated RIP140 protein levels compared with GFP control cells; the protein levels were calculated relative to actin levels (Figure 1A). Real-time RT-PCR analyses revealed similar reductions in downstream metabolic transcription factors, including nuclear receptors (ERRα, PPARα, βand PPARγ) and transcriptional factor NRF-1 genes in a dose-dependent manner (Figure 1B). It is worth noting that ectopic expression of RIP140 through adenovirus at MOI of 20 leads to the induction of coactivators PGC-1α. Additionally, the protein expressions of PGC-1α were inconsistent with the changes in mRNA levels (Figure 1A). Consistent with the above results, the transcriptional level of fatty-acid β-oxidation genes, such as ACADS, ACADM, and HADHB, were also clearly reduced in ARPE-19 cells infected with AdRIP140 (Figure 1D). In addition, we found that glucose transporters 1 and 4 (Glut1 and Glut4) were upregulated in RPE cells in response to RIP140 infection in order to allow glucose uptake to increase (Figure 1C).

Previous results indicated that the transcriptional level of genes involved in lipid metabolism was shown to be altered in AMD [18,26], which was examined. There is an increased expression of apolipoprotein E (ApoE), A (ApoA), B (ApoB), and low-density lipoprotein receptor (LDLR) in RPE cells (Figure 1E).

### 3.2. RIP140 Overexpression Induced Functional Impairments in ARPE-19 Cells

**Figure 2:**
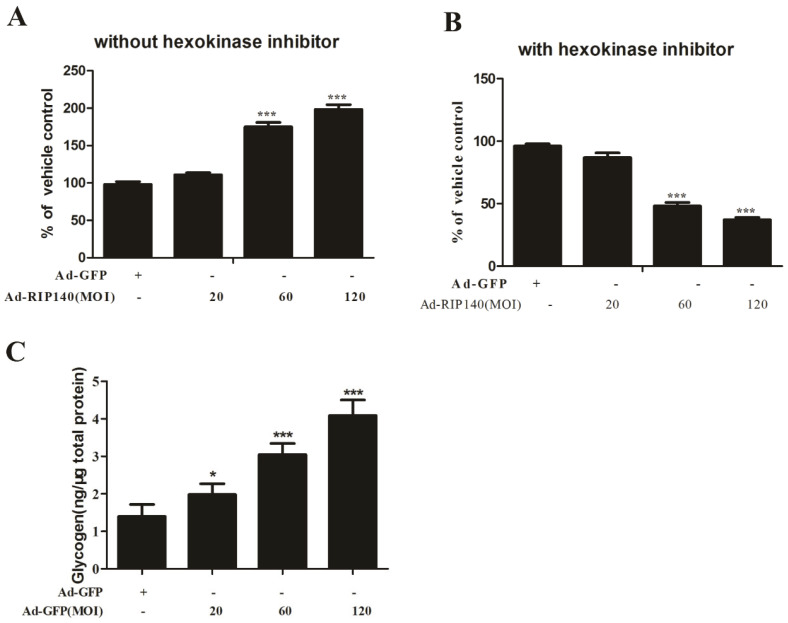
RIP140 overexpressed RPE cells exert lower mitochondrial activity and present higher cytoplasmic glycogen concentration. (**A**,**B**) RIP140 overexpressed RPE cells have significantly lower mitochondrial activity as compared to GFP effected-RPE cells, as indicated by ATP levels measured by a luminescence assay in the absence (**A**) and presence (**B**) of hexokinase inhibitor. (**C**) Measurement of cytoplasmic glycogen accumulation by colorimetric assay showing higher concentration in RIP140 groups compared to control group. * *p* < 0.05, *** *p* < 0.001 vs. Ad-GFP group.

The cellular energy production is clearly affected in AMD by the evidence of measurement of mitochondrial function in RPE cells. Combined with the effects of RIP140 on gene repressing of OXPHOS subunits encoding proteins associated with glucose uptake and lipid processing, it raised the hypothesis that RIP140 regulates mitochondrial function. The mitochondrial activity was evaluated by measurement of ATP production with or without the hexokinase inhibitor, which inhibits the ATP produced by glycolysis. Results indicated that the total ATP production was higher in the RIP140 group without hexokinase inhibitor incubation (Figure 2A), whereas ATP production solely represents mitochondrial ATP production with hexokinase inhibitor incubation, while it was largely reduced in RIP140 transfected ARPE-19 cells compared to GFP transfected RPE cells (Figure 2B), suggesting that the majority of ATP in the RIP140 transfected ARPE-19 cells is produced by glycolysis.

Increased glycogenesis is accompanied by cellular senescence, and massive deposition of glycogen can further exacerbate organ dysfunction [27]. To study whether glycogen accumulation is a cellular phenotype in the RIP140 group, we analyzed the cellular glycogen concentration. It is worth noting that glycogen concentration was significantly higher in the RIP140 group when compared to the GFP group (Figure 2C).

### 3.3. RIP140 Overexpression Upregulated NF-κB Translocation, Subsequent Proinflammatory Gene Expression and Cytokines Release in ARPE-19 Cells

**Figure 3:**
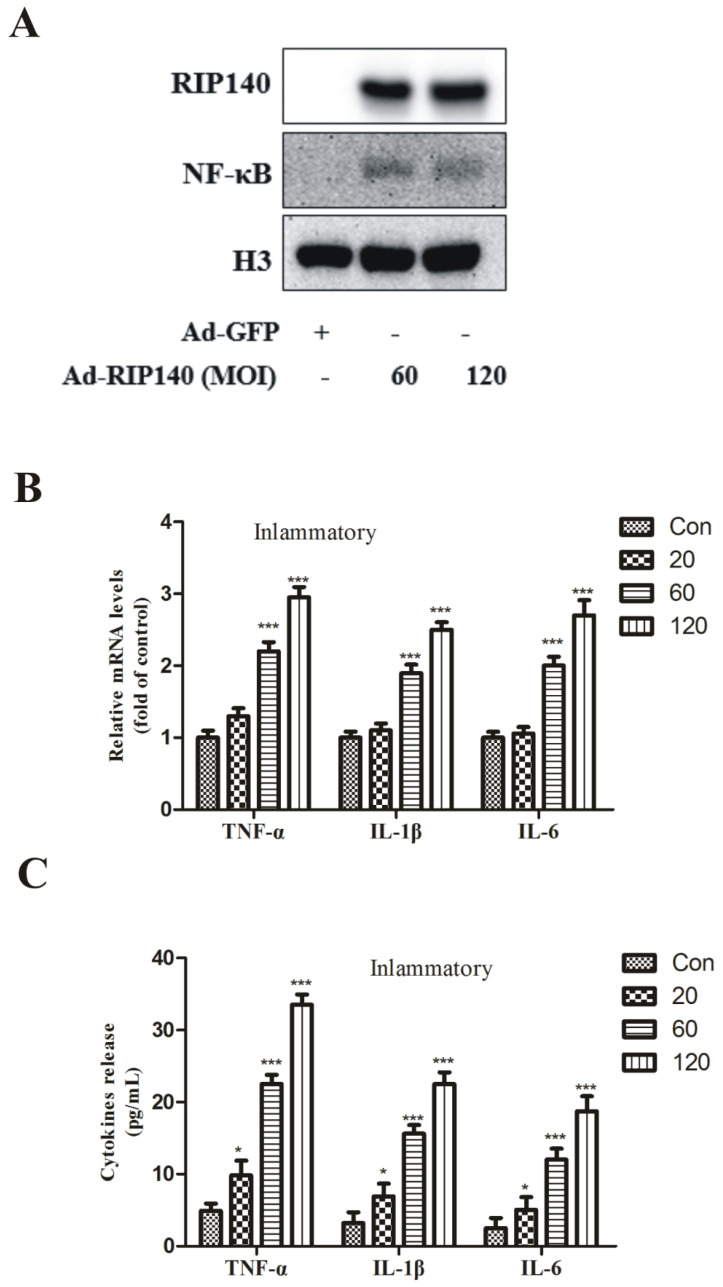
**NF-κB signaling activated following RIP140 overexpression.** RPE cells were infected with adenovirus overexpressing RIP140 (MOI 60, 48 h) or GFP control. (**A**) The translocation of p65. (**B**) TNF-α, IL-1β, and IL-6 mRNA levels were increased in RIP140 groups compared with control group. (**C**) TNF-α, IL-1β, and IL-6 release levels were consistent with the mRNA levels. * *p* < 0.05, *** *p* < 0.001 vs. Ad-GFP group.

Our result suggested RIP140 induced the nucleus translocation of the p65 protein from the cytoplasm (Figure 3A). Therefore, there is a clear upregulation of inflammatory genes such as TNF-α, interleukin-1 beta (IL-1β), interleukin-1 (IL-6) in ARPE-19 cells (Figure 3B) and their release (Figure 3C), which suggested superabundant RIP140 resulted in the formation of a proinflammatory environment in the outer retinal cells.

### 3.4. NF-κB Was Involved in RIP140-Induced Metabolic Dysfunction in ARPE-19 Cells

**Figure 4:**
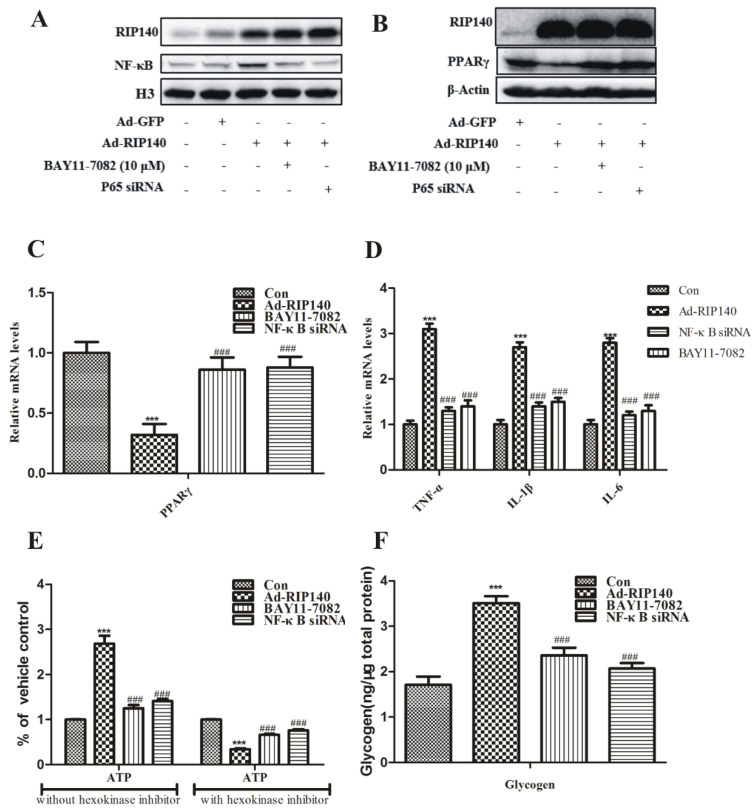
**p65-NF-κB inhibition partially reversed metabolic imbalance by superabundant RIP140.** (**A**) ARPE-19 cells were infected with Ad-RIP140 (MOI 60, 48 h), simultaneously with p65 siRNA or Bay 11-7082 (10μM, 24 h) treatment. (**A**) Nuclear protein levels of NF-κB were assayed by Western blot. (**B**) Protein levels and (**C**) mRNA levels of PPAR-γ, (**D**) secretion levels of TNF-α, IL-β, and IL-6, (**E**) ATP production, (**F**) glycogen accumulation were performed as described in Section 2. *** *p* < 0.001 vs. Ad-GFP group. ^###^ *p* < 0.001 vs. Ad-RIP140 group.

RIP140 could regulate mitochondrial biogenesis through its function as a corepressor for many nuclear receptors, which are crucial controlling factors in metabolism [28]. A variety of transcription factors, such as nuclear receptors PPARγ, have been reported to interact with RIP140 protein to play key roles in regulating metabolic homeostasis [29]. It is generally accepted that PPARγ plays a protective role because many PPARγ ligands can effectively reduce inflammatory processes in vitro and in vivo [2,30]. RIP140 induced NF-κB translocation in ARPE-19 cells was significantly inhibited upon pretreatment with NF-κB inhibitor BAY 11–7082 (10 μM) or NF-κB p65 siRNA (Figure 4A). Meanwhile, RIP140 overexpression reduced the protein and the gene level of PPARγ, which could be partially reversed by p65 interference (Figure 4B,C). To further demonstrate the interactions between RIP140 and NF-κB, we examined the effects of NF-κB inhibition in RIP140-induced inflammation and metabolic dysregulation. Results show that, under RIP140 superabundant condition, induction of inflammatory and repression of mitochondrial function was inhibited upon pretreatment with BAY 11-7082 or NF-κB p65 siRNA (Figure 4D–F).

### 3.5. RIP140-Mediated NF-κB Inflammatory Pathway Involved in Photoreceptor Functional Impairments

**Figure 5:**
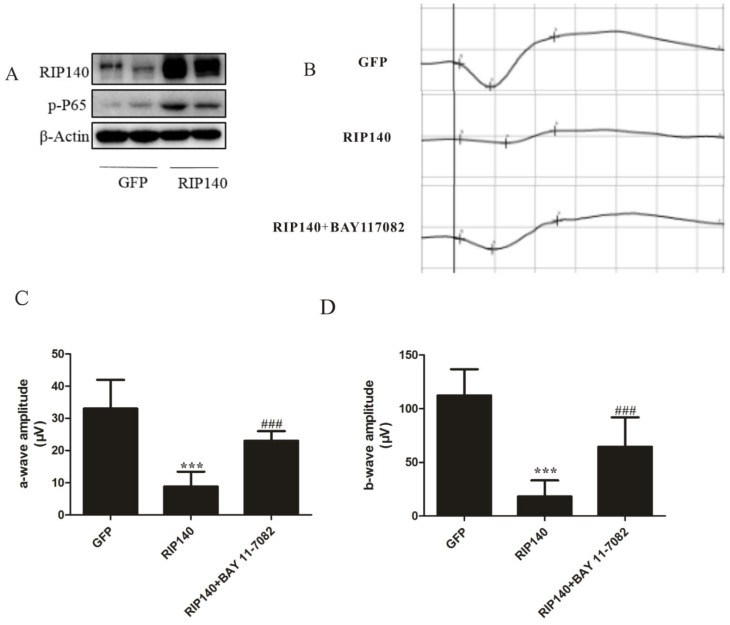
**RIP140-mediated NF-κB inflammatory pathway involved in photoreceptor functional impairments.** (**A**) Western blot showing the expression of RIP140 in the retina 10 days after delivery with adenovirus. (**B**) ERGs from a different group. Amplitude (μV) of (**C**) the a-wave and (**D**) the b-wave. *** p < 0.001, compared with Ad-GFP group. ^###^ *p* < 0.001 vs. Ad-RIP140 group.

Western blot analysis was assessed for adenovirus-mediated expression of RIP140 protein 10 days after subretinal injection. Protein expressions of RIP140 were low in GFP-treated retinas, while highly expressed in RIP140-treated retinas, the phosphorylation levels of NF-κB were also increased simultaneously (Figure 5A). Western blot analysis demonstrated the feasibility of the vector delivery technique. Significant photoreceptor functional impairments were reflected by full-field ERGs performed on GFP or RIP140-treated rats 10 days post subretinal injection (*n* = 6). Amplitude (μV) of the a-wave and the b-wave amplitude of photopic 10.0 ERG in the RIP140 group significantly declined, compared with the GFP group, also there was a clear reduction in a-wave amplitudes of Max reaction compared to the negative control, while NF-κB inhibition partly reversed the reduction (Figure 5B–D). These results demonstrate that inhibition of NF-κB had a beneficial effect on entoretinal function with RIP140 overexpression.

## 4. Discussion

It is well known that there have been no effective treatments for dry AMD until now, and millions of patients lose their vision worldwide each year. PGC-1α plays a major role in mitochondrial biogenesis and oxidative metabolism [31]. It is generally believed that the action of PGC-1α is antagonized by RIP140 in metabolic tissues [32]. NF-κB, the famous transcription factor, regulates many genes involved in inflammation and fatty acid and glucose metabolism [13]. Though RIP140 is a corepressor that downregulates the expression of genes involved in the cellular substrate uptake and mitochondrial β-oxidation, it also functions as a coactivator of NF-κB, promoting the secretion of proinflammatory cytokines in macrophages [12], which suggests there is a crosstalk between metabolic coregulators and inflammation in cells with high energy demand. However, the effects of RIP140 in RPE cells are still unknown.

In this study, after infecting RPE cells with low MOI RIP140, the gene and protein expression of RIP140 was in accordance with its opposing functional cofactors PGC-1, but it is hard to observe changes in their regulators (ERRs, PPARs) and their downstream target genes. Therefore, we hypothesized that a compensating upregulation of PGC-1 may be provoked to set off the effects mediated by RIP140 at the lower level. Moreover, RIP140 negatively regulated key nuclear receptors, transcription factors, as well as their target genes associated with mitochondrial OXPHOS. Moreover, the glucose uptake was also negatively controlled by RIP140 in a dose-dependent manner (Figure 1A–D).

Lipid metabolism is strictly regulated, and its dysfunction could induce excess lipid accumulation within the RPE and Bruch’s membrane [33]. It is well known that RPE cells could secrete lipoprotein-like particles containing apolipoprotein E (ApoE), A (ApoA), and B (ApoB) on the basal side of the epithelium, and the homeostasis of cholesterol is dysregulated in the RPE and adjacent Bruch’s membrane of AMD [34]. More than 40% of lipids in drusen are secreted by RPE [35]. A characteristic of AMD is protein- and lipid-rich lesions in the Bruch’s membrane, and the abundance of ApoE and its cargo, cholesterol, in drusen. Several studies indicate that the material that forms drusen, including ApoE, is secreted from the RPE [18]. The RPE is mainly involved in the formation of basal deposition and drusen. The lipoproteins accumulated from apolipoprotein B100 (apoB100) appear first in the basal deposit and drusen formation. In response to the excess lipid, RPE cells secret the lipoproteins into the systemic circulation [36]. Whereas AMD is accompanied by the dysfunction of the RPE and the formation of deposits of proteins (many related to the complement system) and lipids basal to RPE, it always appears in the form of drusen [37]. The soft drusen and an atherosclerosis-like progression are caused by the lipid cycling pathways in the sub-retinal pigment epithelium basal lamina space [38]. Our results showed that the genes associated with lipid cycling pathways were increased in ARPE-19 cells overexpressing RIP140 (Figure 1E). The metabolic changes in RPE may induce lipid accumulation and thicken the Brunch’s membrane, limiting glucose delivery to photoreceptors.

The function of RPE cells will be affected in the early stages of all forms of AMD, reducing mitochondrial and glycolytic metabolic capacity, then the mitochondrial dysfunction can cause a reduction in mitochondrial oxidative and phosphorylation activity [39]. It is reported that mitochondrial dysfunction in the RPE is a key event in AMD pathology and the mitochondrial damage potentially leads to reduced ATP production. Dysfunction of RPE mitochondria is a characteristic of AMD, which not only induces a decrease in retinal bioenergetics but also disrupts intracellular calcium homeostasis and mitochondria-nuclear signaling, causing a wide range of impacts on cell health [40]. The usability of glucose is closely related to the dysfunction of RPE with retinal degeneration in both animal models and AMD patients [35]. We, therefore, measured mitochondrial activity in Ad-RIP140 and Ad-GFP-transfected ARPE-19 cells. Our data revealed decreased ATP production by mitochondria and increased ATP production by glycolysis in RIP140 superabundant cells compared to GFP-infected cells (Figure 2). These data suggest that RIP140 overexpression mimicked the aging process in RPE cells. The RPE cells are reducing oxidative phosphorylation and meeting their energy demands by increasing glycolysis. These results indicated that superabundant RIP140 reduced ATP production. RPE began to rely on glycolysis to maintain the energy requirement of cells, thereby reducing the glucose flow to the photoreceptors. These results suggest that mitochondria or energy metabolism is an effective target for AMD therapy.

Persistent inflammation response is considered another factor underlying AMD. Chronic low-level inflammation and complement activation have a key impact on the development of drusen. Increased oxidative stress, decreased proteostasis, and gradually increased dysfunction are some of the stress factors that cause inflammation in aged RPE cells. From the results of isolated drusen material, both traditional and newly discovered signaling systems, such as NF-κB and the inflammasome pathways, were significantly activated; these are the proof of proinflammatory activation [41]. Mitochondria from IPS RPE cells derived from AMD patients were significantly damaged, which is indicated by the ATP production reduction and glycolysis enhancement [11]. We speculated that the dysfunction of RPE cells embolism was associated with NF-κB mediated inflammatory pathway and found that overexpression of RIP140 promoted the translocation of NF-κB (Figure 3A) induced proinflammatory gene expression and cytokine release in ARPE-19 cells (Figure 3B,C), which could be reversed by NF-κB inhibition (Figure 4D). Furthermore, RIP140-mediated repression mitochondrial function was weakened in the presence of inhibitors of NF-κB (Figure 4E,F). These results indicate that NF-κB played a key role in RIP140-mediated energy metabolism and proinflammatory response in RPE cells and provide results for the crosstalk between metabolic dysregulation and proinflammatory processes in the AMD progression.

Functionally, PPARs regulate the activity of genes involved in many processes, such as lipid homeostasis, glucose regulation, immune regulation, cell differentiation, inflammation, and wound healing, and are also associated directly with AMD [42]. Moreover, PPARγ is the crucial component of photoreceptor degeneration and vision loss because PPARγ is overexpressed in the retina, specifically in the RPE and choroidal vascular endothelial cells [43]. Our results also show that PPARγ involved in the metabolism and inflammation process induced by RIP140 in RPE cells (Figure 4B,C).

The main feature of dry AMD is the loss of function of RPE cells and the loss of a series of rod photoreceptor cells. The detailed mechanism of the link between AMD and RPE degeneration is poorly understood. Existing evidence shows that the metabolic level of the retina is closely coordinated in a metabolic ecosystem [44]. Subretinal injection of a virus is used when transgene expression is required in the retinal pigment epithelium (RPE) or the photoreceptors [45]. RPE cells constitute a common denominator in AMD disease, regulate the cytokine milieu in the outer retina, and interact with overlying photoreceptors, underlying Bruch’s membrane and choroidal vasculature. Given the above reasons, a therapy aimed at strengthening ailing RPE cells might be useful for AMD treatment [46]. We used subretinal administration of recombinant adenovirus to overexpress RIP140 in the outer retina. Full-field ERG recording is a massed potential from the whole retina [47]. It is reported that the injury of retinal pigment epithelial attenuated the entoretinal function by measuring the a-wave and b-wave of ERG [48]. A decrease in functional activity, as measured by electroretinography (ERG), is also observed in AMD patients and the desired outcome [49]. In our study, metabolism changes in the RIP140 overexpression group were paralleled by functional vision defects. RIP140 treatment induced a reduction of spatial visual function measured by a decrease in the amplitude of the a-wave and b-wave (Figure 5C–E), which confirmed that RIP140 overexpression mediated energy metabolism and proinflammatory response damaged the function of RPE mimics the phenotype of AMD. These results solidly indicate that RIP140 plays an important role in the metabolism function of RPE, which also is required for the development of normal visual ability.

In summary, this study shows that overexpression of RIP140 resulted in the activation of NF-κB and the subsequent increase in cytokine expression and changes in metabolic-associated genes and mitochondrial function. Revealing this complicated signaling pathway could provide knowledge of the pathophysiology of proinflammatory response interacting with energy metabolism changes in the progression of dry AMD. In view of these collective findings, it is likely that therapeutic strategies that modulate the activity of the coregulator of proinflammatory response and energy metabolism and their relationship could have clinical utility for the treatment of AMD in terms of preventing RPE degeneration and create a new path for targeted drug development for the treatment of this intractable neurodegenerative disease of the visual system.

## Data Availability

The data presented in this study are available in the submitted article.

## Supplementary Materials

The following Supporting Information can be downloaded at: https://www.mdpi.com/article/10.3390/cimb44110393/s1, Table S1: Primer Sequences for real-time PCR.
