# Supplementary material for: RIP140-Mediated NF-κB Inflammatory Pathway Promotes Metabolic Dysregulation in Retinal Pigment Epithelium Cells
Source: Curr Issues Mol Biol. 2022 Nov 21;44(11):5788–801. doi: 10.3390/cimb44110393 (PMC9689634; doi:10.3390/cimb44110393)
Supplement: Supplementary file 1 [file cimb-44-00393-s001.zip › cimb-2014050-supplementary.pdf]

Table S1 Primer Sequences for real-time PCR

Gene Symbol	Gene Name	Forward Sequence (5'–3')	Reverse Sequence (5'–3')
ATP5O	ATP synthase subunit O	TTTGAATCCCTATGTGAAGCGTT	CCTTGGGTATTGCTTAATCGACC
ACADM	Acyl-CoA dehydrogenase, C-4 to C-12 straight chain	ATGCCCTGGAAAGGAAAACT	AACCTCCCAAGCTGCTCTCT
ApoA1	Apolipoprotein A1	GAGACTGCGAGAAGGAGGTC	TCTCTGCCGCTGTCTTTGAG
ApoB	Apolipoprotein B	GAG AAA CTG ACT GCT CTC AC	ATGATAGTGCTCATCAAGACT T
ApoE,	Apolipoprotein E	GTTGCTGGTCACATTCCTGG	GCAGGTAATCCCAAAAGCGAC
ACADM	Acyl-CoA dehydrogenase, C-4 to C-12 straight chain	ATGCCCTGGAAAGGAAAACT	AACCTCCCAAGCTGCTCTCT
COX4I1	Cytochrome c oxidase subunit 4 isoform 1	GCACTGAAGGAGAAGGAGAAG	AACCGTCTTCCACTCGTTC
COX5B	Cytochrome c oxidase subunit 5B	GGAAGACCCTAATTTAGTCCCCT	CCAGCTTGTAATGGGCTCCAC
ERRα	Estrogen-related receptor alpha	TATGGTGTGGCATCCTGTG	GTCTCCGCTTGGTGATCTC
GAPDH (housekeeping)	Glyceraldehyde-3-phosphate dehydrogenase	CGACCACTTTGTCAAGCTCA	AGGGGTCTACATGGCAACTG
Glu1		AACTCTTCAGCCAGGGTCCAC	CACAGTGAAGATGATGAAGAC

Glu4	Glucose transporter isoform 4	CTTCGAGACAGCAGGGG TAG	AGGAGCAGAGCCACAGT CAT
HADH B	Hydroxyacyl-CoA dehydrogenase/3-ketoacyl-CoA thiolase/enoyl-CoA hydratase (trifunctional protein), beta subunit	CTTGCTCCGAGAGGGAGT C	AGCTCGTAGCTGGGAGG AAC
IL-1β	Interleukin-1β	CAACAGGCTGCTCTGGG ATT	GGGCCATCAGCTTCAAA GAAC
IL-6	Interleukin- 6	ACTCACCTCTTCAGAACG AATTG	CCATCTTTGGAAGGTTCA GGTTG
LDLR	Low density lipoprotein receptor	CTGGACCGGAGCGAGTA CAC	TGGGTGCTGCAGATCATT CTC
NRF1	Nuclear respiratory factor 1	GCTGATGAAGACTCGCCT TCT	TACATGAGGCCGTTTCCG TTT
PGC-1 α	Peroxisome proliferator-activated receptor gamma, coactivator 1 alpha	GTCACCACCCAAATCCTT AT	ATCTACTGCCTGGAGACC TT
PPAR α	Peroxisome proliferator-activated receptor alpha	ATCGAATGTAGAATCTGC GGG	TCGCACTTGTCATACACC AG
PPAR β	Peroxisome proliferator-activated receptor beta	CCAACAGATGAAGACAG ATGCA	CTGAACGCAGATGGACC TCTA

PPAR α	Peroxisome proliferator-activated receptor gama	AGGCGAGGGCGATCTTGACAG	GATGCGGATGGCCACCTCTTT
TNF-α	tumor necrosis factor-α	GCCCATGTTGTAGCAAACCC	GGACCTGGGAGTAGATGAGGT
